# The malignant transformation of retrorectal cystic hamartomas with blood irregular antibodies positive: A case report: Erratum Urachal adenocarcinoma that metastasized to breast was misinterpreted as primary breast mucinous carcinoma: A rare case report and literature review: Erratum

**DOI:** 10.1097/MD.0000000000005672

**Published:** 2016-12-09

In the articles “The malignant transformation of retrorectal cystic hamartomas with blood irregular antibodies positive: A case report”^[1]^ and “Urachal adenocarcinoma that metastasized to breast was misinterpreted as primary breast mucinous carcinoma: A rare case report and literature review”,^[2]^ which appeared in Volume 94, Issue 49 and Volume 95, Issue 35, respectively, of *Medicine*, a university affiliation was originally given incorrectly. Jinan University should have been reported as University of Jinan in both articles.

## References

[1] Zhao X-R Gao C Zhang Y The malignant transformation of retrorectal cystic hamartomas with blood irregular antibodies positive: A case report. *Medicine*. 2015 94:e2253. 26656372 10.1097/MD.0000000000002253 PMC5008517

[2] Zhao X-R Gao C Zhang Y Urachal adenocarcinoma that metastasized to breast was misinterpreted as primary breast mucinous carcinoma: A rare case report and literature review. *Medicine*. 2016 95:e4612. 27583877 10.1097/MD.0000000000004612 PMC5008561

